# Optimising access to employment support for people with multiple sclerosis through charities: a qualitative study using Normalisation Process Theory

**DOI:** 10.1186/s12913-026-15129-2

**Published:** 2026-07-14

**Authors:** Blanca De Dios Perez, Caolan Senior, Roshan Das Nair, Denise Kendrick, Nikos Evangelou, Ian Newsome, Kate Radford

**Affiliations:** 1 Centre for Rehabilitation and Ageing Research, University of Nottingham, Nottingham, UK; 2 NIHR Nottingham Biomedical Research Centre, Nottingham, UK; 3 Mental Health & Clinical Neurosciences, University of Nottingham, Nottingham, UK; 4 Nottinghamshire Healthcare Trust (Institute of Mental Health), Nottingham, UK; 5 Health Division, SINTEF, Trondheim, Norway; 6 Centre for Academic Primary Care, Lifespan and Population Health, University of Nottingham, Nottingham, UK; 7 Lay Co-author, York, UK; 8 Centre for Rehabilitation and Ageing Research, School of Medicine, University of Nottingham, Room B1387, D Floor, Queens Medical Centre, Nottingham, NG7 2RD UK

**Keywords:** Vocational rehabilitation, Job retention, Multiple sclerosis, Interviews, Third sector

## Abstract

**Background:**

Vocational rehabilitation (VR) interventions aimed at optimising job retention could help people with multiple sclerosis (MS) stay employed longer and prevent premature job loss. Currently, there is a lack of specialist services providing these types of interventions in the United Kingdom, and existing healthcare services are overstretched, hindering the introduction of a new service. This study aims to report on the adaptation of an MS-specific VR intervention for implementation within charities supporting those living with MS.

**Methods:**

Semi-structured interviews with people with MS, employers, and employees from charities, and informal communication with stakeholders from national charities supporting employment for people with disabilities. This study used the ADAPT guidance and normalisation process theory (NPT), which together informed the design, stakeholder engagement, and data analysis using framework analysis.

**Results:**

We conducted semi-structured interviews with 26 participants (11 people with MS, 12 MS charity employees, and three employers), and informal conversations with six stakeholders [charity employment specialists (*n* = 3), disability advisors (*n* = 2), charity CEO (*n* = 1)]. Four themes mapped to the core NPT constructs were identified to explain the implementation process, including service structure, training non-specialists, intervention components, and service promotion. Recommended adaptations include providing guidance on structured intervention sessions, collaborations with external organisations, and monitoring intervention delivery and performance to maximise the use of the limited resources available.

**Conclusion:**

The adaptations identified can facilitate the delivery of the VR intervention within charities. Providing mentoring support, upskilling staff, and strategic collaborations are essential for the long-term sustainability of the intervention in this new context.

**Supplementary Information:**

The online version contains supplementary material available at https://doi.org/10.1186/s12913-026-15129-2.

## Background

In the United Kingdom (UK), 8.7 million people are not working due to a health condition that impacts their work ability, a 41% increase (approximately 2.5 million) over the last decade [1, 2]. The number is also expected to rise by a further 317,000 by 2026 [3]. Additionally, the number of people off sick in the UK has increased significantly over the last few years, with a report from the Chartered Institute of Personnel and Development reporting an average of 7.8 days of sick leave per employee per year [4]. These levels of sick leave incur a significant cost to employers and put people at risk of job loss, stressing the need to optimise employment outcomes for those living with illnesses and disabilities. Therefore, improving access to employment support is particularly needed now more than ever before.

Multiple Sclerosis (MS) is a chronic, often progressive neurological condition [5]. People are usually diagnosed with MS in the prime of their working lives, and many have difficulties remaining in employment [6]. One systematic review (153 studies across 29 countries) found that the pooled overall effect size for unemployment and early retirement was 35.6% and 17.2%, respectively [7]. Another study found that MS affected productivity at work in 77% of the patients [8] – demonstrating the magnitude of the problem. Primary reasons for dropping out of work include common symptoms of MS (e.g., cognitive difficulties and fatigue) and the unpredictable course of the disease [9, 10].

Work is important for the functioning of wider society, but is also beneficial for individuals and can lead to improved health outcomes [11]. For people with MS, remaining in paid employment is associated with improved clinical outcomes such as mobility, fatigue, and mood [12]. For this reason, supporting return to work or job retention has been considered an outcome for health interventions to improve patient outcomes [13]. One such type of intervention is Vocational rehabilitation (VR), aimed at supporting people with illness or disabilities to remain, return or find new employment [14].

Support available to help individuals with MS in the UK to maintain employment is limited and frequently overlooks the nuances and unpredictability of the condition [15]. Because of service limitations (e.g., lack of staff or funding), VR services only meet around 10% of the needs of those with long-term neurological conditions [16] and there is inadequate support for individuals with MS to remain in work [17]. VR can be delivered within the National Health Service (NHS), which provides diagnosis and care to people with MS. However, research has found that NHS waiting lists, the length and frequency of healthcare appointments, and patients’ expectations that the NHS is there to support “healthcare” but not “employment” are key challenges to implementing VR support within existing NHS services for people with MS [15].

In hospitals, VR interventions are delivered by Occupational Therapists (OTs) and psychologists [18]. Our team developed a job retention VR intervention to support people with MS at work and tested this programme within existing NHS services by training a clinical OT [19]. Significant service restructuring and resources were needed to provide this support at a larger scale [19]; therefore, this is likely to be unfeasible to deliver or sustain.

However, given that VR practitioners can have a wide range of professional backgrounds, and these interventions can be delivered in different sectors, such as employability or counselling services [20], this raises the question of whether third-sector organisations (TSOs, e.g., charities) could offer employment support to people with MS.

TSOs offer a wide range of services and support that overcome the limitations of the support provided by existing health and social care services [21]. People with MS have reported that they first seek support from these organisations before raising their issues with healthcare professionals [15]. However, unlike regulated healthcare services, TSOs may have inconsistent standards and protocols across regions. Nevertheless, staff from TSOs could be trained to deliver interventions in contexts with limited resources. Training non-specialists to deliver complex interventions is known as task-shifting, which can address service gaps caused by staffing issues, including within the NHS [22]. While there is some evidence of TSOs delivering evidence-based interventions in the context of healthcare conditions (e.g., HIV [23], and addiction [24], this has not been explored in MS.

Therefore, our innovation is an initial exploration of how TSOs could offer employment support for people with MS, based on adapting our findings from our NHS-based VR research [14], literature on task-shifting, and input from patient and public involvement (PPI) representatives.

The MSVR intervention, previously described elsewhere [19, 25]. The intervention was designed to be delivered remotely over a period of three months and includes a series of core components, such as structured vocational assessment, goal setting, and identification of reasonable adjustments, which are central to the intervention’s underlying mechanisms and need to be preserved across settings. There are also certain components that need to be delivered by specialists, such as legal rights advice, employment advice, and clinical management of MS symptoms. Other aspects of the intervention can be adapted to the charity or community setting, such as the mode of delivery or the staff involved in providing support.

## Methods

This study aimed to determine how to adapt a job retention VR intervention for people with MS to be delivered within MS charities. Specifically, we explored (1) existing services offered by MS charities, (2) barriers and enablers to delivering VR through MS charities, and (3) adaptations to the intervention content, manual, and training package needed in preparation for a future trial.

### Study design

We conducted semi-structured interviews following a post-positivist approach, which assumes an objective reality but recognises researcher biases [26], to explore the views of potential end users and service providers.

### Participants

People with MS, employers (e.g., line managers, human resources staff, occupational health representatives), and MS charities employees were recruited through purposive and convenience sampling by emailing key groups such as PPI networks, employer networks and MS charities. Recruitment stopped when we reached data saturation, which was achieved when no new ideas appeared in the interviews [27].

The inclusion criteria for MS participants were (1) a diagnosis of MS and (2) being in paid employment (including self-employment) or having left the workforce within the last 12 months due to MS or the nature of their job role. Employees of MS charities were eligible if they provided information or advice directly to individuals with MS as part of their role. Employers were eligible if they had experience in supporting someone with MS or other health conditions at work. For all participants, the inclusion criteria included (1) adults aged 18 and over, (2) residing in the UK, (3) able to give informed consent, and (4) able to communicate in English.

Informal conversations were also held with relevant stakeholders such as employment specialists, disability advisors, and staff from TSOs that had experience in designing services to support people with illness or disabilities to remain or return to employment.

### Data collection and analysis

We developed an interview topic guide for each participant group, informed by the ADAPT Guidance [28] and Normalisation Process Theory (NPT) [29] (supplementary file A). Briefly, ADAPT is a consensus-informed guidance that proposes systematic processes for adapting interventions to new contexts and helps clearly report on what does or does not work. ADAPT was selected to identify the key questions required to understand the similarities between the context of the NHS and MS charities. NPT is a social process and implementation science theory that enables researchers to understand and articulate the cognitive and social processes individuals and collectives within a context undertake that are necessary for implementation to occur [30]. NPT was selected because it allows for identifying issues affecting the implementation of MSVR within different contexts and understanding how an intervention can be embedded as part of routine care.

Participants received a participant information sheet explaining the purpose of the research before their involvement and completed a consent form before data collection.

The researcher conducting the interviews (BDP) is a woman working as a Senior Research Fellow with a PhD in Psychology and extensive experience in VR and MS.

Data were collected via Microsoft Teams or telephone according to the participant’s preference. The participants completed only one interview and did not know the researcher conducting the interviews.

Informal conversations were held at an early stage of the study by BDP with six stakeholders [group virtual meeting (*n* = 3), email discussion (*n* = 2), virtual meeting (*n* = 1)], following an unstructured approach to gather contextual insights from their organisation, and explore stakeholders’ perspectives on the topic. These conversations provided preliminary insight that supported the researchers’ understanding of the context of national charities before conducting the interviews. Participants were aware of the academic purpose of these discussions and agreed to participate; however, they were not treated as formal research interviews, and no identifiable data or direct quotations from these conversations were included in the study findings. Notes were taken during each conversation to capture the key points discussed. The participants were alone during the interviews, and the transcripts were not returned to them for feedback. The researcher took notes during the interviews to support the data analysis and interpretation.

Interviews were audio-recorded via telephone, and audio and video were recorded for those completing the interview via Microsoft Teams. The interviews that were conducted via telephone were transcribed by BDP, and Microsoft Teams automatically transcribed the video recordings. Data were analysed using Excel and collated into tables based on the changes needed to refine the intervention.

The data were analysed using framework analysis [31]. Two researchers (BDP, CS) independently coded the interview data and then met to synthesise it. Following Ritchie and Spencer [32], we undertook the following steps: (1) Familiarization: The research team familiarised ourselves with the data by listening to audio recordings and/or reading the transcripts, and discussing observations emerging from this process. BDP and CS led this process but invited discussions from the wider team. (2) Identifying a framework: We had an a priori framework developed based on our interview schedule informed by NPT, so developed a matrix for each theme to explore the relationship between themes [31]. (3) Indexing: Data were loosely fitted to this framework, with data that could not be coded onto the framework was coded under “other” and iteratively revised to synthesise the data accordingly [31]. (4) Charting: We created summaries of the data from each participant pertaining to each theme based on our framework, keeping close to the data and using verbatim text where this was relevant. (5) Mapping and interpretation: We reviewed the framework charts and tried to identify patterns in relation to our research question, and attempting to create a narrative synthesis of the data [31].

The preliminary themes were then presented to a patient and public involvement (PPI) representative (IN) for review and to address any discrepancies identified between the researchers’ coding of the data. The preliminary themes were also presented to the research team before writing up to refine them further.

Changes to the intervention identified through participants’ feedback were categorised by the researchers, analysing the interview data (BDP, CS), according to the underlying reason for change and the priority for implementation using the MoSCoW (Must, Should, Could, or Won’t) framework. The MoSCoW prioritisation was used to guide decisions regarding which changes should be incorporated, considered, or not progressed in the refinement process. The reason for change category captured the rationale for modifying or retaining intervention components, with changes classified as important (participants’ needs, preferences, or barriers considered essential to address), easy (changes that could be implemented with minimal resources or disruption), repeated (issues or suggestions consistently raised across multiple participants), or no change (intervention characteristics not requiring modification).

This study followed the Consolidated Criteria for Reporting Qualitative Research (COREQ) checklist to report on the key aspects of a qualitative study [33] (see supplementary file B).

## Results

We conducted 26 interviews with 11 people with MS (Table 1), 12 employees from MS charities, and three employers (Table 2). Two interviews were conducted by telephone, and the rest via Microsoft Teams.

**Table 1:** Demographic characteristics of MS participants

MS Participants Characteristics	
	People with MS (*n* = 11)
Age [mean (SD)]	46.7 (10.9)
Women	82% (*n* = 9)
Men	18% (*n* = 3)
Ethnicity*	
Asian	9% (*n* = 1)
White British	82% (*n* = 9)
White European	9% (*n* = 1)
**Relationship status**	
In a relationship	91% (*n* = 10)
Single	9% (*n* = 1)
**Type of MS**	
Relapsing-remitting MS	73% (*n* = 8)
Secondary progressive MS	27% (*n* = 3)
Years with MS [mean (SD)]	12.4 (7.8)
Highest Education Completed**	
A-Levels	18% (*n* = 2)
National Diploma	9% (*n* = 1)
Degree	27% (*n* = 3)
Postgraduate	46% (*n* = 5)
Employment Status***	
Working	82% (*n* = 9)
Self-employed	23% (*n* = 3)
Sick leave	9% (*n* = 1)
Unemployed	9% (*n* = 1)
**Standard Occupational Classification (** ***n*** ** = 10)**	
Level 2 (Professional occupations)	40% (*n* = 4)
Level 3 (Technical occupations)	30% (*n* = 3)
Level 4 (Administrative and secretarial)	30% (*n* = 3)
**Working Hours (** ***n*** ** = 10)**	
Full-time	50% (*n* = 5)
Part-time	50% (*n* = 5)
**Type of Employer (** ***n*** ** = 10)**	
Private	40% (*n* = 4)
Public	60% (*n* = 6)
**Organisation Size (** ***n*** ** = 10)**	
Large (> 250 employees)	50% (*n* = 5)
Medium (50–249 employees)	20% (*n* = 2)
Small (< 50 employees)	30% (*n* = 3)
**Industry (** ***n*** ** = 10)**	
Business	40% (*n* = 4)
Education	20% (*n* = 2)
Entertainment	10% (*n* = 1)
Government	10% (*n* = 1)
Healthcare	20% (*n* = 2)

*Ethnicity categories based on UK census terms

**UK classification; A-level (13 years of education), Diploma (13 years of education); Degree (16 years of education); Postgraduate (17 years of education)

***Employment status is a multiple-choice question where some people may have more than one type of employment

Participants with MS were asked about the most common MS symptoms affecting their work performance. Nine symptoms were reported as impacting their work performance, including fatigue (91%), memory and thinking problems (64%), difficulty walking (64%), pain (36%), muscular weakness (36%), difficulty using hands (27%), tremors (27%), vision problems (27%), and bladder and bowel problems (9%).

**Table 2:** Demographic characteristics of employers & MS charity participants

Participants Characteristics		
	Employer (*n* = 3)	MS Charity Employees (*n* = 12)
Man	67% (*n* = 2)	17% (*n* = 2)
Woman	33% (*n* = 1)	83% (*n* = 10)
**Ethnicity**		
Asian	0	17% (*n* = 2)
White British	0	75% (*n* = 9)
White European	100% (*n* = 3)	8% (*n* = 1)
**Working Hours**		
Full-time	67% (*n* = 2)	75% (*n* = 9)
Part-time	33% (*n* = 1)	25% (*n* = 3)
**Type of Employer**		
Private	67% (*n* = 2)	0
Voluntary	33% (*n* = 1)	100% (*n* = 12)
**Organisation Size**		
Large (> 250 employees)	33% (*n* = 1)	42% (*n* = 5)
Small (< 50 employees)	67% (*n* = 2)	58% (*n* = 7)

Informal conversations were held with six stakeholders (four men, two women), including charity employment specialists (*n* = 3), disability advisors (*n* = 2), and the CEO of a charity (*n* = 1).

### Qualitative findings

Four themes and fourteen sub-themes were extracted from the interviews (Table 3). The interviews lasted between 10 and 55 min (average 47 min). The interviews with two participants with MS lasted 10 min, which was shorter than expected because they were not fully engaged in the discussion, potentially due to fatigue and cognitive difficulties. However, their data were included in the analysis because they were rich and discussed several points relevant to the study.

**Table 3:** Coding tree

Theme	Sub-Theme
Service structure and implementation	Existing services available
	Workforce issues
	Personal qualities of staff
	Resource constraints
	Collaborations and partnerships
Preparing for intervention delivery	Training non-specialists
	Intervention assessment and structure
	Addressing gaps in knowledge and support
Intervention components	Goal setting
	Provision of peer support
	Employer engagement
	Information about legal rights
Service promotion	Need for employment support
	Reaching out to the right people

### Theme 1: Service structure and implementation

Both people with MS and employees from MS charities expressed the importance of support services from MS charities to help people with MS manage their condition and ask questions about MS and work.

#### Existing services available

Existing services from MS charities offer high-level information rather than personalised advice. This allows them to deal with a high volume of calls from people requesting support from these organisations. The theme of employment is recurrent, and MS charities organise podcasts and webinars to make the information more accessible to those living with MS.

Employees from MS Charities believed that work is essential for people with MS, and there is a high demand to offer support and advice on this topic.

Interviewer: Do you think there is value in offering support with employment to people with MS?

Interviewee: 100% because probably one thing the charity doesn’t yet cover is a bit around purpose…I know a lot of people in the [MS charity] network… they’ve had to leave their job, and when they leave their job, they lose that sense of purpose… You lose a bit of yourself, and that won’t help with managing the condition and staying as well and as healthy as you can. (charity employee 12)

One issue discussed was how MS charities could offer MSVR services on an ongoing basis. Currently, MS charities offer one-off support based on information and do not offer case management, which would involve supporting the coordination to access services, planning interventions and monitoring progress.

But you’re right, generally speaking, when people call, they speak to whoever picks up the call, so they wouldn’t phone up and expect to speak to me a second time necessarily. (charity employee 01)

The participants with MS, however, preferred services that gave them advice specific to their work circumstances, as opposed to generic information that can be harder to understand or relate to.

If [the service] it was personal, I think that would probably be really supportive. If we had direct contact face to face, like a Teams meeting or something, and if it was personal for me to be able to access so that I could ask questions, yes

Otherwise, maybe just videos that I can watch in my own time or on a website that would be helpful, or a podcast… I’m definitely a visual person. (person with MS 05)

The employers reflected on the value of an MS-specialist service delivered by charities, especially considering that not all employees have access to occupational health services.

Now about accessibility of occupational health service, it’s limited and the limitations have to do with availability of service when there are not enough occupational health clinicians. But it also has to do with the cost, the culture of the organisation. Some organisations think that they don’t need occupational health and that they can manage themselves. (Employer 01)

Interviewer: Have you ever sought information from national charities?

*Interviewee: Yes*,* quite often. Not only MS*,* but others as well. I think many national charities produce a booklet which gives advice to employers*,* and they’re instrumental. (employer 02)*

#### Workforce issues

MS charity employees believed the intervention needed to be delivered by trained personnel who were not managing routine calls as part of their role. Helpline managers were seen as best placed to manage resources in the organisation and know who would be best suited to deliver the employment support.

I would say it’s probably best to go through the helpline manager for that, and then they would be able to coordinate any sort of that (charity employee 05)

Existing working arrangements (part-time working and rotas) mean that taking time off work to receive the training for the intervention could be difficult and would require managers to explore how to rearrange the MS charity workforce to cover existing services.

Depending on the training, we would need to sort of modify our rota accordingly, but I’m sure that’s something that can be sort of looked at depending on what cover we have in place (charity employee 07)

MS charity employees believed the intervention needed to be implemented with consideration of how it would affect the team’s workload. Because helpline workers are constantly on phone calls or addressing requests from people contacting the organisation, they suggested creating roles with greater flexibility to integrate the intervention within the usual work demands.

Most calls, I would very often hand the call over to one of the three information officers. They have the time because they’re not on the phone constantly. Unlike us, they have the time to stop, research, and get more informed. So maybe there could be a possibility that might work well. (charity employee 01)

#### Personal qualities of staff

MS charity employees had a wide range of soft skills to handle complex conversations developed as part of their face-to-face role engaging with people with MS.

You must have empathy, being able to stay calm, level-headed… being able to listen without sort of interrupting, but also engage with the person. But you know, to show that you’re interested but not talk too much at them, you know … and knowledgeable. I guess the knowledge about MS helps because over the years I’ve built up knowledge and it’s not easy to, you know, it’s a huge topic (charity employee 04)

The skills reported by MS charity employees aligned with what the participants with MS highlighted as the most essential skills for the people providing them with employment support.

I think they need to have a lot of empathy and knowledge. If they know something, they tell you. If they don’t, they will find it out for you. Yeah, it’s the empathy that is very good with [MS charity]; you always feel welcome. (person with MS 11)

#### Resource constraints

All participants discussed that MS charities have limited resources in terms of staff, funding, and access to additional support. They felt that these limitations could hamper staff’s ability to provide in-depth support with employment and deliver the MSVR intervention as intended. Finding time to fit sessions within their workload could be challenging for some of them.

It’s really the admin like the booking the appointment, making sure every appointment is in the calendar, making sure you have time either side of the appointment to kind of prepare and then kind of note anything you do any follow up (charity employee 08)

Small teams, volunteer-led services, high staff turnover, and part-time staff can make it challenging to meet the intervention demands. Employees from MS Charities suggested alternatives to ensure the MS charity and MSVR delivery were always sufficiently staffed.

It may be useful to have maybe two people just in case one is on leave or just so that consistency is there to provide that [MSVR intervention] support. So maybe having these two people in is important (charity employee 02)

The charity employees identified an alternative approach to training existing staff: hiring a designated individual to provide employment support. However, this solution was not without its challenges.

If it were new, a new person or persons, then yeah, that would definitely be a financial implication, but it is how it all works, I know that fundraising can help with staffing… (charity employee 05)

#### Collaborations and partnerships

To overcome some of the barriers identified, participants suggested developing partnerships between charities, legal services, and the Government to provide more comprehensive employment support and fill the existing gaps in the services offered.

*I think that would come out of the training to some extent*,* is sort of not knowing where the key areas of support are and tapping into them*,* whether it’s through access to work or disability law service or whatever. And presumably if the other charities are providing the programme as well*,* interacting between all of those because it’s a good service for people with MS (*charity employee 03)

Collaboration was seen as vital for two areas of the intervention: “support with benefits” and “advice on legal rights”. Both people with MS and MS charity employees reported the importance of understanding legal rights (i.e., UK Equality Act 2010) and how they relate to their working environment.

*I do write letters which say at the bottom*,* which detail the law and the facts*,* and at the bottom say I’m happy for you to share this with your employer*,* so that someone can go look*,* that’s a solicitor. That’s what the law is here. That’s where*,* you know*,* reasonable kind of requests are declined in the first instance*,* and there needs to be a bit of a stronger voice involved… (*charity employee 06)

Informants felt that MS charities were already a source of information and knowledge for occupational health representatives who are tasked with providing support to employees with health conditions they are not familiar with. Therefore, the idea of further developing the relationship between employer organisations and MS charities was welcomed.

*We’re not experts in any particular medical condition*,* but we can bring our background to bear together and understanding of the key issues as they relate to work and then try and put that into non-medical language for managers….(employer 02)*

### Theme 2: Preparing for intervention delivery

This theme encompassed the practicalities that needed to be in place before the staff from MS charities were ready to offer information and advice about employment.

#### Training non-specialists

Although the employees of MS charities believed they possessed experience across a wide range of topics and were consistently seeking development opportunities, they felt that further training would be necessary to deliver MSVR and address issues that require more than a one-off conversation.

I’m always thinking “ah, what this person needs is someone who can work with them over a period of time to help them explore this situation and move forward”. And I’m always looking for employment support programmes, and they just aren’t a lot… if we could say, well, we know we might know about this thing… that could be popular. (charity employee 08)

The key topics identified for the training included recognising key issues at work, understanding legal rights, benefits, employer engagement, and awareness of available services. Since employment is a recurring topic on the helplines, the staff welcomed the idea of the intervention and the opportunity to be trained on such an important subject.

I mean, I think certainly support officers like me would welcome and you know, benefit from having more information, more knowledge, a better understanding of what is out there (charity employee 01)

Offering training was viewed positively because it would assist staff in enhancing their skills and experience in supporting people with MS at work, an area they do not often address.

I’ve only had one client in the three months that’s asked for support in terms of communicating with an employer. So that’s all the experience I have of it and [MS charity] so far. (charity employee 10)

A participant with MS also reflected that employees from MS charities will meet people in moments of distress. Therefore, these employees from MS charities should also be supported to deal with challenging situations.

Perhaps you need something like even a flow chart; say somebody phones with [an issue] this is what you do if somebody phones in if they’re depressed, do XYZ. (person with MS 04)

#### Intervention assessment and structure

MS charities employees reported that assessing a person’s needs at work and structuring the intervention accordingly was a complex task; they could find it challenging to provide sessions on specific intervention topics.

It might be quite hard to provide a structured session on fatigue because I suppose you’d have to tailor it to whether the person was doing a physical job or more cognitive fatigue…I’d be happy to work on something. (charity employee 11)

Most MS charities’ employees were confident in their knowledge and understanding of common MS symptoms (e.g., fatigue, pain, cognition). Participants suggested including ideas for sessions in the training and intervention manual.

Just making sure everyone knows what’s going on and what the intervention is, and how that intervention should be delivered, though really clear process documents about how it should be delivered could be really helpful

Obviously, it will be different for each helpline or organisation, but at least if you start from there, everyone has something to work with about putting it in place. (charity employee 08)

#### Addressing gaps in knowledge and support

Receiving mentoring support or having access to a key contact to address areas where they did not know how to support a person was seen as beneficial to address scenarios they had not faced before.

You would need training on everything… I think when you’re going to drill down into something more specific, even if you know things…there is always going to be that question you think “oh I didn’t think of that” and someone will come along with that question (charity employee 09)

While the support hours were deemed sufficient and feasible compared to more intensive training or mentoring, participants highlighted the need to cover all aspects of the intervention to ensure MS charity employees were qualified to provide employment support. For context, the original intervention included a two-day training and monthly mentoring sessions [19].

I suppose the other thing which would be part of the process would be getting ourselves to a skill level where we can present it [information] in a way that is meaningful to somebody and is actually providing practical support to people in a way that will goes beyond giving them the written information and letting them look through the issues themselves. (charity employee 03)

Because the topic being discussed (i.e., employment difficulties) could be considered sensitive, participants suggested offering a support network to those delivering the intervention to ensure they could request prompt support.

Give them [MS charity staff] almost like an emergency helpline for them because if they get somebody that says I just want to end it all because I’ve got MS, and I can’t feel my legs and I don’t think I’ll ever walk again or like when I lost my sight you know that would be pretty grim. (person with MS 04)

### Theme 3: Intervention components

This theme highlighted that employees from MS charities feel they would greatly benefit from additional training or support around specific topics to ensure they deliver the intervention at the highest quality level.

#### Goal setting

Setting goals is an essential component of the MSVR intervention, and this is not part of the service provision/skills that MS charity employees have. Therefore, this was seen as a key component of the intervention that required additional guidance and training before intervention delivery.

Well, as a helpline information officer, that’s not something that we would do we wouldn’t be setting goals […] Yeah, I think we’d need a heap of help for doing that, because that’s not what we’re trying to do…I’m just thinking, Oh my gosh. (charity employee 09)

#### Provision of peer support

A recurrent idea discussed by participants with MS was the need to incorporate peer support as an intervention component. Participants wanted to learn how others handled difficult situations, such as telling their employer they have MS or pivoting their careers to live their professional dreams. Also, when people with MS reported on positive experiences from previous support they received, engaging with others with MS was seen as one of the most positive aspects of the support:

There were others with MS, and we got to share our advice on how to manage fatigue caused by MS…doing this with other [people] with MS was really really good because they could understand how it feels and give advice to one another. (person with MS 07)

#### Employer engagement

All participant groups highlighted this sub-theme as relevant. People with MS reported the need for a service offering information to their employers, although some were disbelieving that employers would think this was necessary.

Well, they [employers] will say that they know that already. Like the understanding of MS is a general thing for them (person with MS 02)

MS charity employees reported that they already engage with employers but would need further training to offer more comprehensive support.

Maybe some of the social side of things in terms of if the boss or managers are being a little bit difficult… what would be a good approach? Some skills in terms of how to handle a line manager that might be a little bit difficult… Possibly even some training with like the organisation ACAS (Advisory, Conciliation and Arbitration Service)… so that would be good to have a bit more knowledge about that. (charity employee 04)

Employers welcome the idea of MS charities offering them additional support or information. However, they stressed the importance of personalising the information shared with the employer. This is because, in the past, organisations have provided similar information for different employees, and that makes employers less likely to follow recommendations.

I think it’s good that various stakeholders work together, provided, of course, that everyone provides evidence-based advice… It’s often when charities provide advice and support to individuals that they tend to have a very generic approach, so they would give similar advice to everyone with MS they see. But this may not always be correct. If it comes from a charity, they may lack the expertise and the time to understand what their individual needs are (Employer 01)

#### Information about legal rights

Another area where the MS charities’ employees had no expertise or knowledge about was related to support with legal rights.

The only thing I don’t have a huge amount of experience with, but I’m more than happy to research and learn about it, are the actual, specific legal issues surrounding MS in the workplace (charity employee 11)

However, many MS charity employees reported being familiar with other organisations and services that would be suitable to offer legal advice, and they could support the person contacting these organisations.

### Theme 4: Service promotion

This theme covers the need for employment support and TSOs potential role in offering this, notwithstanding inherent challenges, such as targeting the right people to offer and receive support and overcoming barriers to offering in-person support.

#### Need for employment support

For participants with MS, there was a recurrent theme – there is no support available to help them in employment, either from their workplace or healthcare services.

When I was diagnosed, I was kind of in a state of shock, and I just immediately handed them my notice. I felt that more support could have been given to me […] I found it difficult because a lot of places don’t want to employ somebody with a disability, and it would have been quite useful to have that MS and work support. (person with MS 03)

The main barrier to accessing support with employment, apart from the lack of services available, is that people do not know what support is available to help them remain in paid employment. People with MS can also hesitate to ask for support for fear of the perceptions of declined abilities at work. Addressing the concerns and having a positive view about requesting support with employment before they experience difficulties and advertising the service on a wide range of networks can help people with MS access the support they need.

A lot of them [people with MS] are not aware of the support. I think promoting services and putting out there [showing] that there is support… and I think the newly diagnosed webinar will be good even if you do a slide just to say talk about work it will be good (charity employee 02)

People with MS believed that the MS charities could play a key role in supporting them with their needs, given the resource challenges the NHS is facing.

We cannot get a doctor’s appointment very easily at the moment, so I am quite happy if the [MS charity] were able to step in (person with MS 08)

Attending services in person could impact the range of people who end up accessing the services because those living in remote areas, with mobility difficulties, or high levels of fatigue would be less willing to take part in the support with the employment programme. Therefore, all participant groups suggested the need to provide support remotely.

We do offer video calls, telephone calls and emails… They don’t have to come into the centre, so I have clients who I just speak to on the phone that I’ve never met in person (charity employee 10)

#### Reaching out to the right people

Even though there was a perceived need for the service, MS charities’ employees acknowledged that recruiting people with MS for a new service can be complicated until the service becomes established.

I think the initial problem would be the referrals, you know, because it’s a new service. People might often think about whether or not they’d like to approach your service. (charity employee 07)

One approach to resolving this issue is to promote the service broadly via new platforms, such as webinars and social media, which have gained popularity among younger audiences.

Obviously, back in 2009, social media was not that big of a thing. I just say social media now could play a part in advertising, and places like [MS charity], and like [MS charity] could have that. So, it’s just like something that can highlight that they do offer support for people. (person with MS 03)

Reaching out to younger generations and those newly diagnosed was seen as crucial to empower them and avoid a premature workforce exit.

Looking back to when I got diagnosed, I really didn’t know how it was going to work, how it’s what’s going to happen to you. So having somebody to talk to is important (person with MS 09)

Both MS participants and employees from MS charities reported a potential barrier to reaching our target population (i.e., employed people with MS); particularly those who are working with MS and managing their condition well may not reach out to MS charities for support and will not learn about the service availability.

And what I wonder is if there are people out there who have Ms who work, who, because they’re fighting through it and they haven’t reached a point, or at least recognised that they’ve reached a point where they need support. We don’t know they exist because they haven’t said anything yet. They’ve remained silent. (person with MS 01)

### Intervention changes informed by NPT

The interview findings have been summarised into a table of recommended intervention changes mapped to NPT [29] (Table 4).

Regarding the coherence construct (i.e., meaning and sense making by participants), MS charity employees emphasised the difference between their existing role focusing on information provision and the new role in the employment intervention, which will require providing advice and maintaining a continued relationship with individuals with MS who need support. Several training areas were identified, along with the necessity to provide group mentoring sessions to develop a network in which MS charity employees could share their knowledge and experiences. For the successful implementation of the intervention within the MS charities, it must align with the organisational goals.

In terms of cognitive participation (i.e., commitment and engagement by participants), there was a strong focus on assessing staffing levels and the qualifications of available personnel to ensure the effective implementation of the intervention, thereby eliminating gaps in support provision. Additionally, there were recommendations on how to effectively engage key stakeholders, such as employers, to secure their commitment to the intervention and maximise its potential impact.

For collective action (i.e., the work participants do to make the intervention function), participants with MS and employees from MS charities proposed creating employment-specific platforms and communication channels to facilitate the integration of the intervention within existing services. There were also discussions on how to simplify the intervention manual and resources to enable MS charity staff to provide employment support without depending on clinical reasoning.

Finally, for reflexive monitoring (i.e., participants reflect on or appraise the intervention), MS charity employees suggested approaches to monitor whether and how the intervention works through existing data collection platforms and targeted qualitative data collection to identify areas for improvement, thereby facilitating implementation.

**Table 4:** Intervention changes mapped to Normalisation Process Theory

NPT Construct	Components	Changes / Recommendation	Reason for Change	MoScoW Priority
**Coherence** (i.e., Meaning and sense making by participants)	Differentiation	Employees from MS charities should clearly report to people accessing their services that a new employment support service is available, emphasising the distinction from existing services.	IMP	Should do
		Develop materials (webinars, brochures, adverts) highlighting intervention benefits	Easy	Could do
		Existing services in charities provide generic information. To differentiate MSVR from existing services, the employees from MS charities need to provide individualised support with employment.	No change	Should do
		MSVR needs to complement existing services by providing a “needed” area of support, and limit involvement in areas where there could be duplication from existing services available (e.g., benefits advisors, legal rights).	REP	Must do
	Communal specification	Safety net: Employees delivering the intervention should have the telephone number of the research team to contact them in case of need.	IMP	Must do
		Provide group training to the employees from MS charities to develop relationships with others who have a similar role. Key areas of training identified include identifying intervention needs and structuring intervention, goal setting, types of discrimination under the Equality Act 2010.	REP	Must do
		Offer group mentoring sessions to develop relationships between the employees from MS charities delivering the support, allowing them to share their experiences and approaches to overcoming difficulties.	Easy	Should do
	Individual specification	Clearly outline the responsibilities and expectations of the employees from MS charities during the intervention delivery process.	IMP	Must do
		Provide employees from MS charities a clear intervention pathway and create standard operating procedures (SOPs) on the intervention components.	REP	Could do
		Offer individual mentoring session to those MS charity employees who are struggling with confidence.	Easy	Could do
	Internalisation	Highlight the importance of employment support to improve the wellbeing of people with MS.	IMP	Could do
		Write case studies and success stories about people with MS who have received employment support and as a result they have achieved positive employment outcomes.	Easy	Could do
		Align the intervention to the MS charity goals to support its adoption.	IMP	Should do
**Cognitive participation** (i.e., Commitment and engagement by participants.)	Initiation	Identifying key members of staff within the MS charity that would be suitable to delivering employment support.	REP	Must do
		Develop an internal referral pathway so that employees from MS charities working on different areas of the organisation can signpost people to the new employment programme.	Easy	Must do
		Develop networks of collaborations between charities, employer organisations, and healthcare service to support the intervention delivery.	IMP	Could do
		Secure funding through fundraising, grants or corporate sponsorship to support the long-term sustainability of the programme.	IMP	Must do
	Enrolment	Recruit and train at least two member per MS charity to support the continuity of the service due to high levels of staff turn around.	REP	Must do
		Facilitate the liaison of the MS charity employment specialist with specialist in other areas (e.g., legal support, benefits application, coaching, trained volunteers) to reduce the burden of the intervention on MS charity staff.	REP	Could do
	Legitimation	Gather evidence on the effectiveness of the intervention to create case studies on people who have successfully manage employment challenges due to the support received from MS charity employees.	REP	Must do
		Develop relationships with employers to ensure this support is seen as “needed” instead of a “burden” and reinforce the need to support employees with disabilities at work.	IMP	Must do
		Disseminate findings from the intervention through key stakeholders.	IMP	Must do
	Activation	Develop an implementation plan including intervention structure, timelines, and responsibilities.	REP	Should do
		Develop a peer mentoring scheme where people with MS support each other discussing employment challenges and how they overcame them.	IMP	Could do
**Collective action** (i.e., the work participants do to make the intervention function)	Interactional workability	Support the integration of the intervention within existing services from MS charities by allowing the use of multiple channels of communication to deliver the support.	REP	Should do
		Develop a platform for people with MS to access support from the intervention without the need for external referrals.	IMP	Could do
	Relational integration	-	-	-
	Skill-set workability	Develop an intervention manual and training set for MS charity employees to develop knowledge on employment challenges, reasonable adjustments, and symptom management in the context of work.	IMP	Must do
		Simplify manual and provide examples of activities and topics to address during the intervention to reduce need for clinical reasoning and help MS charity employees to structure sessions.	REP	Must do
	Contextual integration	Collect data on employment needs and support requested by people with MS from existing software used by the MS charities to collect data from services.	IMP	Must do
		Support people with MS in accessing alternative services such as MS specialist or employment services to address areas beyond the reach of the intervention.	IMP	Could do
**Reflective monitoring** (i.e., participants reflect on or appraise the intervention)	Systematisation	Monitor participant’s progress throughout the intervention, dropouts (reasons if provided) and changes in outcomes of interest.	IMP	Must do
	Communal appraisal	Explore the views of employees from MS charities on the impact of the intervention on people with MS and their employers, and barriers to the long-term sustainability of the intervention.	IMP	Should do
		Integrate feedback on employment progress and needs within the software used by charities to record service use.	IMP	Could do
	Individual appraisal	Collect feedback (quantitative and qualitative) to assess acceptability and understand the impact of the intervention on employment from the perspective of people with MS and their employers.	IMP	Should do
	Reconfiguration	Develop a working group to identify issues with the intervention and further training needs of staff to adapt and modify the intervention (or its delivery) based on learned experience.	IMP	Should do

NPT: Normalisation Process Theory. REP: Repeated; IMP: Important. MoScoW Prioritisation: Must have; Should have; Could have; Won’t have

## Discussion

In this study, we explored how a VR intervention to help people with MS remain in paid employment can be adapted for delivery by charities supporting them. There was consensus among the different participant groups, who believed there was value in providing employment support services for people with MS through MS charities, particularly given the lack of such services. To do so, our study identified the key training needs of staff, structural changes to TSOs to implement the intervention and approaches to maximise existing resources by developing relationships with key stakeholders such as employers and disability organisations. The NPT constructs helped us understand the findings by examining how stakeholders understood the intervention, engaged with it, and anticipated the work required for implementation.

Although there was consensus among participants about the benefits of incorporating employment support services within TSOs, they identified two key areas where extensive service modifications were needed.

First, the major difference between how MSVR should be delivered and existing services from MS Charities refers to the continuity of support. Currently, MS charities only provide information, usually as a one-off instance, and do not provide advice. For the VR programme to be implemented within these organisations, there was a need for continued support, booking appointments in advance, and monitoring progress. This could be a barrier to implementation due to the unpredictable workload and limited staff available to offer support.

The second area of concern referred to the training needs of MS charities’ employees. Although the employees from these organisations reported knowledge of MS and its symptoms due to engaging with multiple people with MS and working on an MS-specific charity, there were also concerns about key topics such as legal rights, reasonable adjustments, and structuring interventions. Therefore, training staff before implementation was considered essential and could overcome the skill gap needed to offer employment support. Training nonspecialists to deliver complex healthcare interventions has been widely used in other areas, such as reducing family violence [34], providing psychotherapy for mood difficulties [35], and managing alcohol use [36], amongst other conditions.

However, if the MSVR intervention becomes a longer-term service embedded within a TSO, further financial resources would be needed to recruit, train, and retain staff. Indeed, a systematic review (of 31 studies; [37]) of barriers and facilitators to implementing interventions by TSOs identified resource limitations, in particular staff and finance, to be the most reported barriers. Although this review did not include MS-related TSOs or UK studies, it is interesting to note the similarities between barriers and facilitators identified in both studies.

As also highlighted in the aforementioned review, organisational culture and alignment with its vision/mission were seen as facilitators. In our study, the MS charity employees welcomed the idea that the MSVR intervention included a support system, such as mentoring sessions to assist them in problem-solving challenging scenarios that people with MS might bring to the sessions and guide them to deliver the intervention as intended. MS charity employees had positive views about receiving this type of support in a group setting to understand the experiences and skills that employees from different organisations had. Evidence suggests that mentoring within the context of a clinical trial enhances therapists’ confidence and intervention fidelity [38]. The fact that VR support could potentially be incorporated into the charity’s service offerings is another facilitator for implementation.

Two key aspects of the VR intervention adapted for this study are: (1) empowering the person with MS to identify key issues at work and (2) supporting them with symptom self-management [25, 28]. These aspects have been found in previous research to be essential to improve employment outcomes in MS [39]. Interestingly, our participants with MS also highlighted the need for peer support as part of the intervention and learning about success stories from others with MS who are still at work. Peer support interventions have been used to improve function and participation in multiple long-term conditions such as spinal injury [40] and cancer [41]. In MS, a pilot trial in Australia evaluated a peer support in a group setting focusing on providing employment and found that these interventions are acceptable to people with MS [42]. Future research could explore the impact of individualised peer support and its effect on employment outcomes for people with MS.

Lastly, a recurring idea during the interviews was that a job retention programme aligns with the organisational views of MS charities, as employment has consistently been reported as an area of interest for people with MS. Ensuring the intervention aligns with the organisation’s current goals and vision has often been identified as a facilitator for implementation leading to successfully integration of the new intervention within existing services in the host organisation [43].

### Strengths and limitations

This study has multiple strengths. A key strength is that we followed the ADAPT guidance [28] to support the intervention adaptation and followed NPT [29] to frame the findings reporting on factors that may hamper implementation. This guidance has been used for relatively similar purposes to adapt interventions from high-level to resource-constrained contexts, for example, adapting an intervention for children with autism to be delivered in South Asia [10].

The limited number of employers recruited for the study is a limitation. We could not recruit a larger sample despite contacting multiple organisations and targeting the study advertisements to employers. This reflects a potential real-world challenge in implementing such a VR intervention that includes employers. However, the employers recruited provided a rich description of their views and experiences that had thematic relevance, which helped us shape our findings. Some of the interviews were brief, but they offered some rich data that we could incorporate in our analysis. Finally, it could be argued that most of our respondents were positively minded towards VR and the importance of TSOs in providing such services. However, we recruited from more than one TSO and respondents were not reluctant to share negative or contrary views.

### Implications for practice and research

This study illustrates areas where employees from MS charities are willing and could be upskilled to offer employment support. Providing training covering key areas such as legal rights, identifying reasonable adjustments, and structuring the intervention support could be beneficial to provide the first level of support to people with MS. Developing relationships with external organisations such as employment services and healthcare teams could enable MS charities to signpost those with complex issues to specialist services.

Future research could attempt to deliver such a VR intervention in MS-related TSOs and assess the acceptability and feasibility of delivery of the intervention, adapted based on the results of this study.

## Conclusion

People with MS lack access to VR services that can help them remain employed. Existing MS charities bridge the gap between healthcare and employment services by offering information through helplines and websites. However, the service provision is limited to generic information.

Employees from MS charities can be upskilled to offer support with employment to people with MS. Key aspects to consider for successful implementation include the provision of training and mentoring to support intervention delivery, supporting staff to change their mindset from information provision to case management and developing relationships with external organisations to provide support in areas beyond the remit of the VR intervention.

## Supplementary Information

Below is the link to the electronic supplementary material.

Supplementary Material 1

## Data Availability

The datasets used and/or analysed during the current study are available from the corresponding author on reasonable request.

## Abbreviations

MS: Multiple sclerosis
NHS: National health service
OT: Occupational therapist
VR: Vocational rehabilitation
NPT: Normalisation process theory
