# Supplementary material for: Optimising access to employment support for people with multiple sclerosis through charities: a qualitative study using Normalisation Process Theory
Source: BMC Health Serv Res. 2026 Jul 14;26:1374. doi: 10.1186/s12913-026-15129-2 (PMC13651039; doi:10.1186/s12913-026-15129-2)
Supplement: Supplementary file 1 [file 12913_2026_15129_MOESM1_ESM.docx]

**Supplementary File A - Interview Topic Guide**

[Please note: This is a semi-structured topic guide that is designed to be used flexibly with each participant. As such, the questions and prompts (presented as sub-questions) asked in each interview are likely to vary slightly.]

**Introductory statement**

I am a researcher at the University of Nottingham working on a project that aims to understand what can be done to support people with Multiple Sclerosis (MS) to remain at work.

Vocational rehabilitation (VR) is a process whereby those disadvantaged by illness or disability can be enabled to access, maintain, or return to employment or other useful occupations. It can help people, return to work following periods of absence due to illness and help those currently working but experiencing difficulty to remain in work. These types of support are known as job retention interventions. VR can also help people progress in their careers despite illness or disability and support them in withdrawing from work when they can no longer continue.

We have previously developed a job retention VR intervention for people with MS and tested this in the NHS. Today, we would like to ask you some questions about how this needs to be modified to fit within services provided by national MS charities that offer support to people with MS in the UK.

[Share MSVR Intervention Description v1.0 10.02.2024]

Your name will not be mentioned on any published documents, and any names or places you might refer to will be anonymized when transcribed. All information you provide will be kept confidential and only used to develop our understanding for research purposes.

# **Preliminary questions MS Participants**

1. Have you ever requested support from MS charities?
a) If yes, to which organisations?
b) What support did you request?
c) What support are these organisations currently offering? Who provided the support?
d) Were there any gaps in terms of support provided?

# **Preliminary questions MS Charity**

1. Can you tell me about your role as [insert name]
   a. What support do you provide to people with MS as [insert role]?
   b. How are people referred to you for support?
2. Are there any gaps in the services currently provided by your organisation?

# **Preliminary questions Employer**

1. Can you tell me about your role as [insert name]
2. Do you have experience supporting people with MS in employment?
   a. Do employers follow the recommendations? Are there any challenges you face?
   b. Are there any gaps in the provision of [insert name] in the UK?
3. Have you ever requested information or support from national charities?
   a. If yes, what type of information?
4. How would you feel if you received information or advice regarding the employee’s condition from national charities?

# **Questions from ADAPT Framework**

**Planning for and undertaking adaptations**

1. What changes can be made to overcome the differences between the NHS and the MS charity? [Prompt: staffing levels and skills, funding, knowledge, links with other organisations].
   a. How would you like [insert name of MS Charity] to support you with work?
   b. Are there any limitations to the existing support available?
   c. How could we inform people with MS accessing MS charities services about the intervention?
2. The current intervention has a training package and intervention manual. If we want to deliver the intervention within MS charities, we will need to adapt these resources.
   a. What sort of changes should we include in the intervention manual and training? [prompt: further information on legal rights, more detailed guidance on intervention components, additional training, etc.].
3. What costs and resources should we consider when delivering the intervention within MS charities? [prompt: staffing available, competitive funding, etc].
4. Who is most suitable to deliver the job retention programme within MS charities?
   a. How should they be recruited?
   b. What are the skills/attributes of employees from MS charities?
   c. What would be the training needs of the person recruited?

**Planning for and undertaking evaluation**

5. What information would policymakers at MS charities would like to know about the impact of this programme? [prompt: time of staff to deliver support; benefits reported by people accessing services, etc.]
6. What data are already routinely collected from these organisations that we could use to measure the impact of the programme? [prompt: number of people interested in receiving the service, people reporting employment issues, etc]

**Long-term implementation**

7. What long-term partnerships and capacity will be needed to maintain the intervention?
   a. What are the possible barriers to offering support with employment from MS charities?
   b. What are the possible facilitators to offering support with employment from MS charities?
8. How can we monitor whether the intervention continues to be delivered as intended? [prompt: collect data on session content, record sessions, etc.].
